# APAVAC Immunotherapy for the Adjuvant Treatment of a Canine Mucosal Melanoma

**DOI:** 10.3390/vetsci11120628

**Published:** 2024-12-06

**Authors:** Valentina Rinaldi, Laura Bongiovanni, Paolo Emidio Crisi, Massimo Vignoli, Renato Ennio Peli, Stefano Masci, Andrea Boari, Riccardo Finotello

**Affiliations:** 1 Department of Veterinary Medicine, University of Teramo, 64100 Teramo, Italy; lbongiovanni@unite.it (L.B.); pecrisi@unite.it (P.E.C.); mvignoli@unite.it (M.V.); repeli@unite.it (R.E.P.); aboari@unite.it (A.B.); 2 Department of Biomolecular Sciences, Faculty of Veterinary Medicine, Utrecth University, 3584 CS Utrecht, The Netherlands; 3 Clinica Veterinaria Colli Innamorati, via Colli Innamorati 21, 65125 Pescara, Italy; stefanomasci@hotmail.it; 4 Ospedale Veterinario I Portoni Rossi, Anicura Italy Holding, via Roma 51, 40069 Zola Predosa, Italy; riccardo.finotello@anicura.it

**Keywords:** melanoma, dog, immunotherapy, tumour, oncology

## Abstract

An 11-year-old spayed female Beagle presented with tenesmus and was identified with a rectal wall mass. Diagnostic imaging (abdominal ultrasound and computed tomography) localised the mass in the right rectal wall and documented no evidence of metastatic disease. Subsequently, the dog underwent surgery for tumour excision. A histopathological diagnosis of melanoma was performed. To confirm the tumour histotype, immunohistochemistry was performed using anti-Melan A and anti-Ki67. Neoplastic cells exhibited focal Melan A immunoreactivity and widespread nuclear immunoreactivity for Ki67 with a Ki67 index of 27%. Adjuvant immunotherapy with APAVAC^®^ was initiated. After APAVAC administration, no local or systemic acute adverse events were observed. Four pre- and post-contrast computed tomography (CT) studies were performed in an 18-month follow-up period every 4–5 months. Follow-up rectal palpation and conscious visualisation of the surgical site have also resulted in no macroscopic signs of tumour recurrence. The dog remains alive and with no clinical evidence of tumour recurrence and/or distant progression at the time of writing, therefore, surviving over 540 days from the diagnosis.

## Simple Summary

An 11-year-old spayed female Beagle presented with difficulty defecating and was diagnosed with a mass in the rectal wall. Diagnostic imaging localised the mass in the right rectal wall and documented no evidence of metastatic spread. The mass was surgically excised and identified as a melanoma with features indicative of aggressive biological behavior. Adjuvant immunotherapy with APAVAC^®^ was initiated. After APAVAC administration, no local or systemic adverse events were observed. Serial computed tomography (CT) studies and physical examinations were performed in an 18-month follow-up period to monitor for signs of cancer progression. The dog remains alive and with no evidence of tumour recurrence and/or spread at the time of writing, therefore, surviving over 540 days from the diagnosis.

## 1. Introduction

Melanoma is a malignant tumour that originates from melanocytes and exhibits a wide range of biological behaviours based on the site of origin and histological features [1]. Among melanomas, those of mucosal origin are the most aggressive, being associated with a high rate of local recurrences, systemic metastases, and short disease-free and survival times [1]. Following diagnosis, a complete clinical staging is performed focusing on regional lymphatic drainage and sites of systemic metastases (e.g., lungs, liver, adrenal glands, and kidneys); treatment strategies are typically multimodal, involving surgery, radiotherapy, and systemic therapies [2]. Systemic adjunctive treatments include intravenous platinum compounds, melphalan, and/or temozolomide; however, these have been consistently associated with low response rates and short-lasting effects [3,4,5,6,7]. Therefore, in an attempt to improve the outcomes of canine melanoma patients, over the past two decades, veterinary oncologists have focused on the development of immunotherapy strategies, following the lead of human melanoma research [8]. Liao and colleagues [9] have reported the induction of tyrosinase-specific antibody responses in three of nine dogs vaccinated with human tyrosinase DNA. In 2010, a DNA tyrosinase vaccine (Oncept^®^, Boehringer Ingelheim Animal Health USA Inc., Duluth, GA, USA) was developed for dogs affected by melanoma, but its efficacy remains contentious [10,11,12]. Following on from the dubious benefit of Oncept^®^, a different DNA vaccine directed against chondroitin sulphate proteoglycan 4 (CSPG4) has been trialled, with more encouraging results in the therapeutic management of canine oral malignant melanoma (OMM) expressing CSPG4 as evidenced in two studies [13,14]. However, this DNA vaccine is still not commercially available and only administrable within Italian territory under special licenses. Canine OMM has been recognised to possess unique features in contrast to human cutaneous melanoma as discussed by Prouteau et al. [15]. Nonetheless, canine OMM seems to be an appropriate in vivo model for studying human mucosal melanoma (MM). A recent study indicated the presence of two molecular subgroups within MM that could potentially benefit from specialised treatments, such as immune checkpoint inhibitors or targeted therapies, applicable to both human and veterinary medicine [16,17]. Some methodologies, employed in both human and veterinary cancer treatments, encompass the utilisation of autologous tumour cells and/or their extracts, sometimes combined with bacterial adjuvants as well as genetically altered tumour cells [18,19,20,21], heat-shock proteins (HSP) [22,23], and tumour-specific peptides [24]. It is documented that the use of autologous HSP derived from tumours, linked to small tumour proteins and peptides, forming HSP–peptide complexes (HSPPC), can circumvent immune evasion caused by cancer heterogeneity, immunising the host against a broad repertoire of individual tumour-associated antigens (TAA) [23,25]. Through this mechanism, HSPPC should provide protection against tumours derived from the same clones from which the complexes are purified. Indeed, the entire repertoire of TAAs of a single tumour, including mutated antigens that make each tumour antigenically distinct from another, should be presented and recognised by the patient’s immune system. To enhance the immune system’s antitumor response, hydroxyapatite is used as an immunological adjuvant, aiming to activate cytotoxic T lymphocytes. Additionally, hydroxyapatite has many other advantages: the capacity to purify proteins via chromatography, biocompatibility, attraction of monocytes and macrophages to the implant area, and functioning as a carrier to deliver proteins to APCs [26,27]. APAVAC’s (Hastim, 2022) activity has only been assessed in two human patients with cutaneous melanoma: one patient with lymph node metastases (stage III melanoma) and another with distant metastases in multiple organs (stage IV melanoma). The first patient was disease-free 8 months post-treatment (study closure) although the patient with stage IV disease had progressive disease and died 3 months after starting APAVAC [26]. In veterinary medicine, APAVAC is currently used as part of immunochemotherapy in the treatment of canine lymphoma, suggesting that this may boost tumour response compared to standard chemotherapy, and has not shown adverse effects [28,29]. The authors aim to describe a case of incompletely excised aggressive mucosal melanoma of the rectum, treated with an APAVAC vaccine as a single adjunctive modality.

## 2. Case Presentation

**Figure 1:**
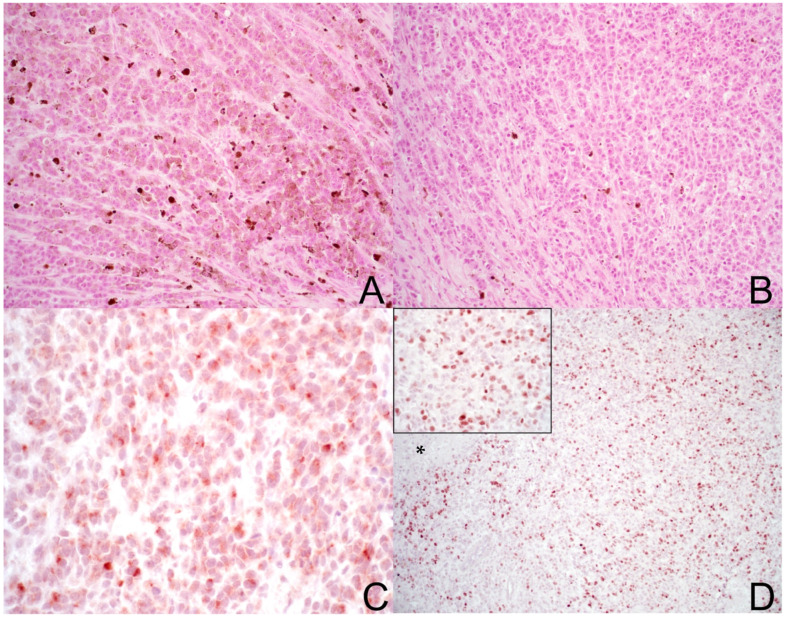
(**A**,**B**) Histological appearance of canine malignant anorectal melanoma. Neoplastic melanocytes are arranged in bundles and sheets. Neoplastic cells with cytoplasmic pigment are more abundant in some areas of the tumour tissue (**A**) while they are scattered in other areas (**B**). (**A**,**B**): 200×, bar: 200 µm; haematoxylin and eosin (H&E). (**C**,**D**) Immunohistochemical staining of canine malignant anorectal melanoma showed neoplastic cells with focal Melan-A immunoreactivity (**C**) and widespread nuclear immunoreactivity for Ki67 ((**D**), inset). * Indicates an area of necrosis. (**C**): 400×, bar: 100 µm; (**D**): 100×, bar: 400 µm; inset 400×, bar: 200 µm; Novared chromogen and haematoxylin counterstain.

A 11-year-old spayed female Beagle weighing 20 kg was presented to the Veterinary Teaching Hospital of Teramo University for tenesmus. During a clinical examination, no significant abnormalities were identified other than a right-sided rectal wall mass; this was causing external deformity, and its landmarks could be identified upon rectal digital examination. A cytological examination of fine needle aspiration (FNA) samples from the lesion was performed, and this was consistent with melanoma. The patient underwent comprehensive blood tests (haematological and biochemical), which revealed no significant abnormalities. Basic staging, including three-view thoracic radiographs and abdominal ultrasound, showed no evidence of metastases. The dog subsequently underwent surgery for tumour excision, and the excised tissue was subjected to histopathological examination. A malignant, multinodular, highly cellular neoplasm was observed on histopathology. Only a very small tract of the rectal mucosa was evident in only one tissue slide, most probably due to extensive tumour ulceration, haemorrhage, and necrosis. The neoplasm primarily expanded and replaced the submucosa, infiltrating the underlying muscular and fibro-adipose tissues. Neoplastic cells were arranged in closely apposed bundles and sheets, sometimes in nests (Figure 1A,B) separated by a fine to moderate, pre-existing fibrovascular stroma. Neoplastic cells were polygonal in shape, with moderate to severe anisocytosis, a high nucleus-to-cytoplasm ratio, and scarce to moderate cytoplasm with indistinct margins, and were sometimes filled with granules of dark brown pigment (melanin). The pigment was not homogeneously present in the tumour tissue, and was more abundant in some areas (Figure 1A) and scarce or absent in others (Figure 1B); pigment was present in about 20% of the tumour tissue histologically analysed. Nuclei were large, round to oval, with one or more evident nucleoli, and there was marked anisokaryosis. Numerous mitotic figures were observed (36 in 10 HPF, 40 X) alongside scattered apoptotic cells. Surrounding and infiltrating the neoplasm at its edges, an abundant lympho-plasmocytic inflammatory infiltration was observed. A diagnosis of canine malignant anorectal melanoma was made based on the histopathological features [30] and anatomical location of the mass. In order to confirm the diagnosis, immunohistochemistry was performed after bleaching of the sections by using heat-induced antigen retrieval in TrisEDTA pH 9.0 and using anti-Melan A (Abcam, Cambridge, UK, dilution of 1:150) and anti-Ki67 (clone MIB-1, Code number: GA626, Agilent-Dako, Glostrup, Denmark, dilution 1:150) antibodies overnight at +4 °C. Neoplastic cells exhibited focal Melan-A immunoreactivity (Figure 1C) and widespread nuclear immunoreactivity for Ki67 (Figure 1D and inset), with a Ki67 index of 27%. The Ki67 index was calculated by means of image analysis on a minimum of five photos (40 X) acquired from intratumoural hotspots. Areas of necrosis and inflammation were avoided. Pictures were taken from non-contiguous hotspot areas where Ki67 appeared to be highly expressed. The count was performed manually by one operator (LB) using the multi-point tool of ImageJ software (Version 1.52a, NIH, Bethesda, MD, USA). The Ki67 index was calculated as the number of positive cells on the total number of counted cells. Negative and positive controls were applied in the immunohistochemical experiments for the two antibodies. APAVAC was purified from melanoma specimens and prepared as a vaccine. The method of preparation is described in detail in a datasheet from the manufacturer (https://www.hastim.fr/en/animal-health/apavac-kit accessed 6 December 2024). Once prepared, the doses were stored frozen at −20 °C until use. The dog received a total of eight doses of the vaccine (0.5 mL intradermal injection) on weeks 1, 2, 3, 4, 8, 12, 16, and 20. Radiotherapy was discussed but this was declined due to logistic issues, and no other treatments were, therefore, administered. After APAVAC administration, no local or systemic acute adverse events were observed, and the owners were instructed to monitor the inoculation site and dog’s demeanour and to report any abnormality; no late, acute or chronic, adverse events were reported throughout treatments and the follow-up period. Four pre- and post-contrast computed tomography (CT) studies were performed in an 18-month follow-up period every 4–5 months, with a slice thickness of 1.25 mm (S1). CT scan results did not document the development of regional or distant metastases but the persistency of a millimetric heterogeneous thickening of the right para-anal tissue, likely consistent with scarring sequelae. Follow-up rectal palpation and conscious visualisation of the surgical site have also resulted in no macroscopic signs of tumour recurrence. The dog remains alive and with no clinical evidence of tumour recurrence and/or distant progression at the time of writing, therefore, surviving over 540 days from the diagnosis.

## 3. Discussion

In the field of small animal medicine, there is a growing awareness of the escalating frequency of malignant tumours [31]. Notably, the occurrence of tumours in dogs exceeds 1000 instances per 100,000 dog-years [32], a rate surpassing that observed in humans. Similar to humans, the likelihood of tumour development in dogs rises with increasing age. Malignant tumours account for approximately one-third to one-half of all mortalities in dogs, predominantly affecting aged individuals [33]. Traditionally, the approach to treating malignant tumours in dogs has evolved by mirroring the methodologies used in human treatments, leading to trials of various immune system-based therapies [33]. This approach is influenced by the anticipated outcomes of immunotherapy observed in humans. Since the discovery of cancer antigens in 1991 [34], extensive research has been conducted in human medicine on cancer vaccines, particularly to those based on peptides [35]. Nevertheless, such peptide-based cancer vaccines have not seen application in canine medicine due to insufficient identification of specific antigens targeted by the vaccine in various canine tumours. Additionally, analysis of dog leukocyte antigen (DLA) across different breeds, which is crucial for pinpointing effective peptides for each dog, has not become a standard practice [33,36]. To address this limitation, a plasmid DNA vaccine was created that expresses the full length of a tumour antigen, thereby eliminating the need for DLA and peptide analysis. Among the limited antigens recognised in canine tumours, tyrosinase was chosen as a target for OMM in dogs, paralleling its use in human and mouse melanomas [37]. Therapeutics targeting tyrosinase were subsequently sanctioned for OMM treatment in dogs before their approval in human medicine (Bergman, 2010). Oncept^®^ is a plasmid DNA xenogeneic vaccine incorporating the human tyrosinase gene, and emerged from this line of research. Its initial efficacy was demonstrated in mice in 1998 [38], leading to numerous clinical trials in canine OMM [9,39]. These trials culminated in the vaccine’s approval in 2010 as a supplementary treatment, alongside surgery and radiation, for stage II and III canine OMM. Although many subsequent trials have been conducted, its efficacy remains debated [40]. This instance illustrates the use of clinical trials on naturally occurring canine tumours to inform and expedite drug approval for human use, though this specific DNA vaccine is yet to receive authorisation for human application [41]. Given the relatively higher prevalence of OMM in dogs as compared to MM in humans, the prospect of evaluating the use of DNA vaccines and immune check point inhibitors on canine OMM patients could be useful as another treatment option for dogs as well as providing precious help in providing valuable information for treating MM in humans [36]. In cases of cancers that are uncommon in humans but prevalent in dogs, implementing novel treatments in dogs first could reduce development costs by enabling faster proof-of-concept studies. In veterinary medicine, rectal localisation of melanoma is extremely rare. A recent case report has been published describing two cases from a histopathological perspective [30]. Primary malignant anorectal melanoma in humans, which is a rare and aggressive cancer originating from melanocytes at the anal dentate line or rectal mucosa [42,43], has no known causes or predisposing factors. Typical clinical manifestations include anorectal bleeding, pain, and tenesmus, often accompanying an anorectal mass [42], mirroring observations in the dogs discussed in the current report. The prognosis in humans is generally poor, primarily due to late-stage diagnosis [42,44]. At initial diagnosis, up to 60% of human patients already exhibit metastases to regional lymph nodes [44,45]. Similarly, primary or metastatic anorectal melanoma is extremely uncommon in dogs, with just a few cases reported in veterinary literature [30,46,47], and very little is known about prognosis; it was, therefore, challenging to predict tumour behaviour in our case. Histopathology prognostic parameters for OMM (nuclear atypia, mitotic count, and Ki67 index threshold) are usually applied to melanomas of other mucosal locations. In the present case, we observed high nuclear atypia (>30%), a high mitotic count (>4/10 HPF), and a high Ki67 index (27%), which are the main markers of poor prognosis. However, the real prognostic significance of these parameters is still unknown when applied to mucosal melanocytic tumours of locations different than the oral cavity [1]. Furthermore, we also observed an invasive growth and deep invasion that have been significantly negatively correlated with the survival of dogs with melanocytic neoplasms from various body sites [1]. Multifocal areas of necrosis were also reported in the present case. The presence of necrosis has been significantly negatively associated with survival for several tumour sites (oral cavity, lip, feet, and skin) [48] but has not always been confirmed in other studies, and is difficult to assess and of unknown specific significance for anorectal melanoma. The degree of pigmentation is of uncertain prognostic significance [1] even in cutaneous and oral melanocytic tumours; however, the distribution of pigmented cells in the reported case (around 20%) was similar to that reported in previous cases of anorectal melanoma in both dogs [30,48] and humans [42]. This is why, due to its rarity and in contrast to well-documented canine cutaneous and oral melanoma, predicting the behaviour of gastrointestinal melanoma in dogs is challenging [1]. Further research with more cases is needed to define the clinicopathological characteristics and prognostic indicators for malignant anorectal melanomas in dogs. Nevertheless, it is not possible to rule out that the dog could have remained disease-free regardless of the use of adjunctive treatments; although, the tumor histological features and incomplete surgical excision would make this possibility less likely. It is the authors’ opinion that this result lays the groundwork for exploring the therapeutic role of APAVAC in the adjunctive treatment of aggressive canine melanoma. Supplementary Figure S1 could be seen in the supplementary file.

## Data Availability

Raw data can be made available upon reasonable request.

## Supplementary Materials

The following supporting information can be downloaded at https://www.mdpi.com/article/10.3390/vetsci11120628/s1: Figure S1: Post-contrast CT image. Transverse view of the descending colon/rectum approximately 2 cm from the anus demonstrates asymmetry of the rectal wall. There is mild thickening of the right side (arrow), corresponding to the area of prior surgical intervention.
