# Supplementary material for: APAVAC Immunotherapy for the Adjuvant Treatment of a Canine Mucosal Melanoma
Source: Vet Sci. 2024 Dec 6;11(12):628. doi: 10.3390/vetsci11120628 (PMC11680214; doi:10.3390/vetsci11120628)

Figure S1: Post-contrast CT image. Transverse view of the descending colon/rectum approximately 2 cm from the anus demonstrates asymmetry of the rectal wall. There is mild thickening of the right side (arrow), corresponding to the area of prior surgical intervention.

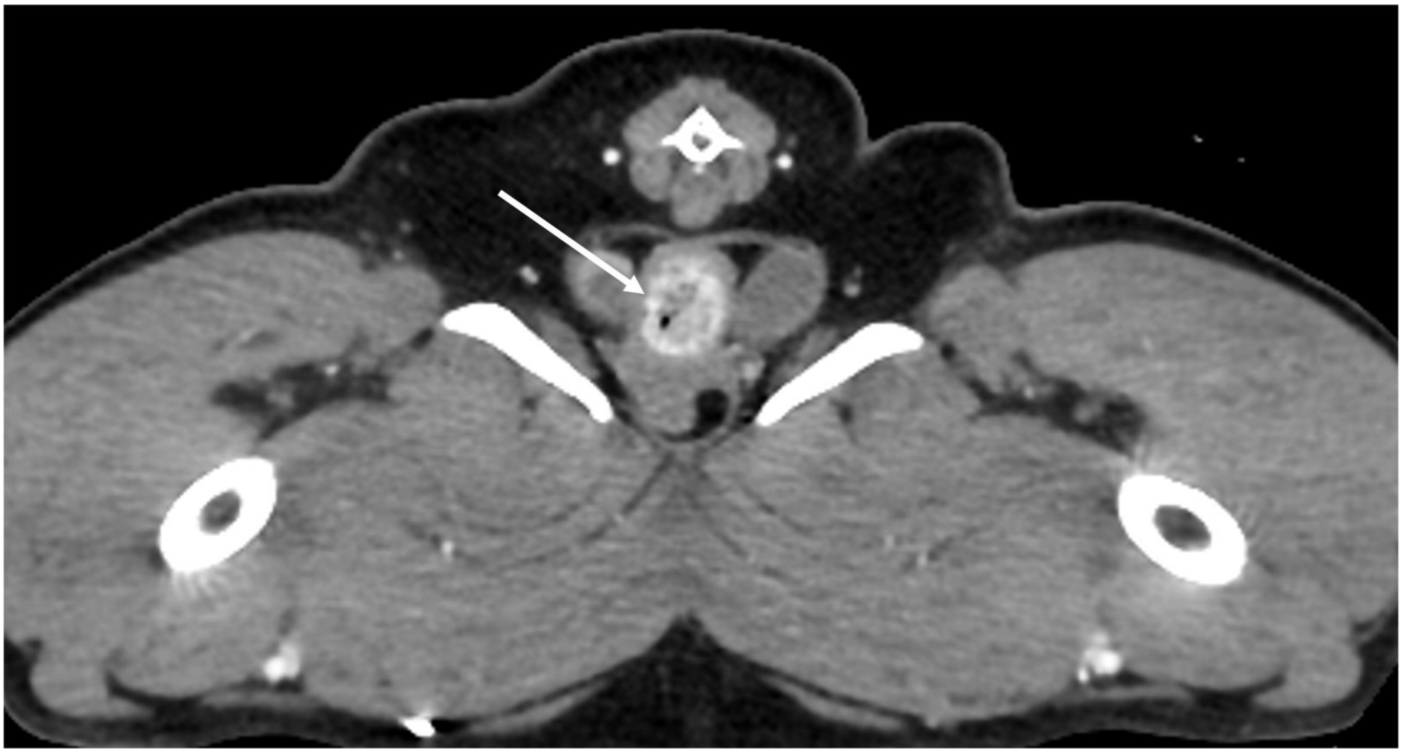

Supplement: Supplementary file 1 [file vetsci-11-00628-s001.zip › vetsci-3267294-supplementary.pdf]
